# Alteration in regional tissue oxygenation of preterm infants during placement in the semi-upright seating position

**DOI:** 10.1038/srep08343

**Published:** 2015-02-09

**Authors:** Anna Petrova, Rajeev Mehta

**Affiliations:** 1 Department of Pediatrics, Rutgers Robert Wood Johnson Medical School, New Brunswick, NJ 08901

## Abstract

We investigated whether the cerebral (rSO_2_-C %) and renal (rSO_2_-R %) tissue oxygenation of preterm infants is altered by repositioning from the supine to semi-upright position for pre-discharge car seat testing. Near-infrared spectroscopy was used to measure rSO_2_-C and rSO_2_-R, which were recorded simultaneously with vital signs in 15 preterm infants for 30 minutes in supine, 60 minutes in the semi-upright (at 45 degrees in a car seat), and 30 minutes in the post-semi-upright (supine) position. Changes in rSO_2_-C and SO_2_-R were mostly within 1 Standard Deviation (SD) of baseline mean levels in the supine position. Decrease in rSO_2_-C and rSO_2_-R (more than 1SD below baseline mean) was recorded in 26.7% and 6.6% of infants respectively, which persisted even after adjustment for variation in heart and respiratory rate, and pulse oximeter measured oxygen saturation (P, 0.0001). Re-positioning the infants from the car seat to supine position was associated with normalization of the rSO_2_-C. Alteration in rSO_2_-C and rSO_2_-R in a car seat was independent from the gestational and post-conception age, weight and presence of anemia. We concluded that approximately one-third of preterm infants show minor reduction of cerebral tissue oxygenation in the semi-upright (car seat) position.

Maintaining optimal oxygen supply of regional tissue during the neonatal period remains a challenge in the care of preterm born infants^1^,^2^. Body positioning is a recognized factor that may affect cardiorespiratory functioning and oxygen parameters of preterm born infants. Some studies reported that the prone position is advantageous for respiration and oxygenation as compared to supine or lateral in oxygen-dependent ventilated preterm infants^3^,^4^. However, other investigators could not find any significant effect of supine or prone positioning on distribution of ventilation in both, ventilated and spontaneous breathing preterm newborns^5^. Similarly, body positioning (supine, prone, lateral or prone head elevated), was not found to reduce apnea, bradycardia, or oxygen saturation in spontaneously breathing preterm infants with apnea^6^. Data regarding concordance of infants' body positions with oxygen levels in different tissues are also inconsistent. A study in stable preterm infants reported an almost 5% decrease in cerebral tissue oxygen content during a head-up 30 degree tilt^7^ whereas another found no effect of this degree of head elevation on the cerebral and mesenteric tissue oxygenation^8^.

It has been shown that the 45 degree semi-upright position used for pre-discharge car seats testing^9^ is associated with the risk for development of apnea, bradycardia, and periodic breathing^10^,^11^. Therefore, it would be relevant to investigate whether cerebral and renal tissue oxygenation of clinically stable preterm infants is altered by repositioning from supine to the 45 degree semi-upright position in a car seat. To answer this question, we compared near-infrared spectroscopy (NIRS) measured cerebral and renal tissue oxygen saturation and simultaneously recorded hemodynamic and respiratory parameters with respect to the infant's body position: (i) supine (baseline); (ii) 45 degree semi-upright (in a car seat); and post-semi-upright (after return to the supine position).

We hypothesized that changing the body position from supine to the 45° degree semi-upright may alter cerebral and renal tissue oxygenation in preterm born infants. The American Academy of Pediatrics (AAP) recommends pre-discharge testing of cardio-respiratory function during placement in a car seat for all infants born at less than 37 weeks^12^. Knowledge regarding any alteration in regional tissue oxygenation during the car seat challenge would be important for further understanding and improving the criteria for the assessment of results of the car seat test.

## Results

Among the 16 subjects, one infant with congenital hypothyroidism was excluded from the final analysis. Gestational age and birth weight of the studied infants varied from 25 to 36 weeks and 800 to 2250 grams, respectively (Table 1). Approximately 20% of the infants had been mechanically ventilated during the NICU admission but all were clinically stable at the time of enrolment with no evidence of bradycardia (HR < 90 bits/min) or hypotension (MAP < 30 mmHg). Anemia of prematurity was recorded in 46.7% of the 15 studied infants. Amongst the 21346 total pulse oximeter readings (SpO_2_), more than 98% were in the normal range and a few isolated SpO_2_ (%) readings ranged between 80% and 70% in the baseline supine (0.07%), semi-upright (0.11%), and post-semi-upright supine position (0%).

### Overall analysis of changes in tissue oxygenation

As shown in Table 2, re-positioning of the infants from supine to the 45 degree semi-upright position was associated with decrease in cerebral (rSO_2_-C) and increase in renal tissue oxygenation (rSO_2_-R), which was equivalent to a difference in Z score of less than 0.5 SD from mean levels at the baseline stage. Generally, cerebral tissue oxygenation returned to baseline but renal tissue oxygenation stayed elevated in the post semi-upright (supine) position (Table 2).

### Individual analysis of changes in tissue oxygenation

**Figure 1:**
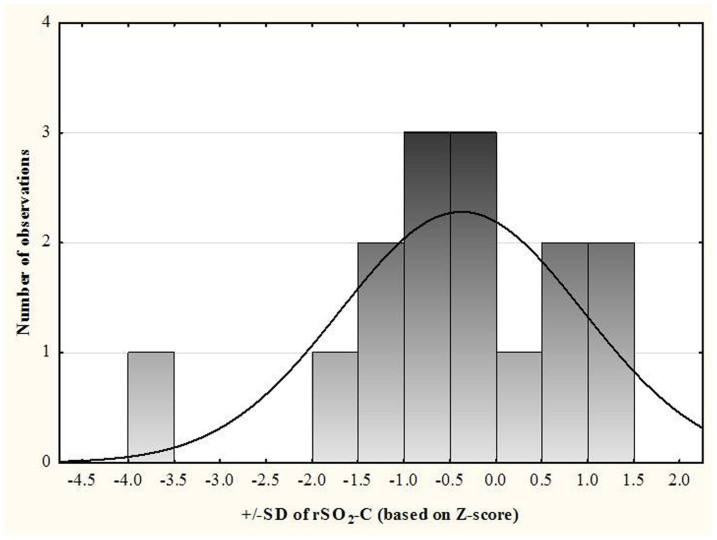
Distribution of SDs of rSO_2_-C in the semi-upright position compared to the mean of rSO_2_-C in the supine position for each participant.

**Figure 2:**
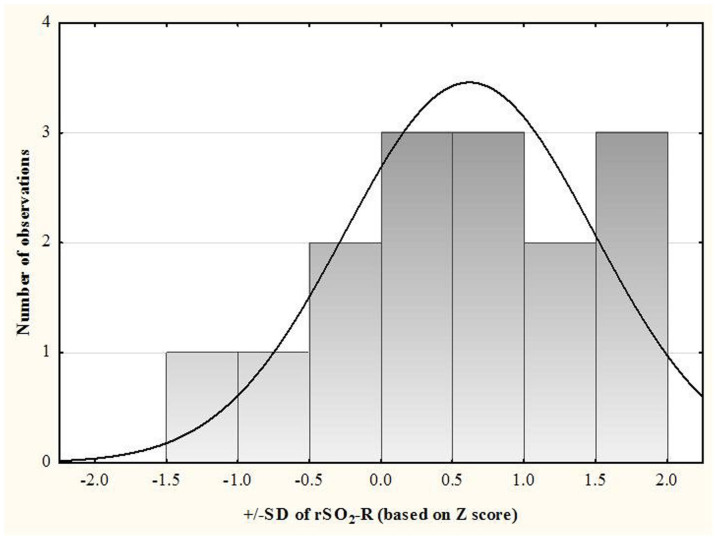
Distribution of SDs of rSO_2_-R in the semi-upright position compared to the mean of rSO_2_-R in the supine position for each participant.

Individual analysis showed that in 60% of infants, changes in rSO_2_-C (%) and rSO_2_-R (%) in the semi-upright position were within +/−1SD of baseline mean, and 26.7% and 6.6% of infants respectively, had rSO_2_-C (%) and rSO_2_-R (%) levels more than 1SD below the mean in baseline supine position (Figure 1 and Figure 2). Concordant decrease in rSO_2_-C (%) and rSO_2_-R (%) levels of more than 1SD was recorded in one infant, who was born vaginally at 36 weeks with birth weight of 2075 grams, was not intubated, did not show evidence of anemia of prematurity, and was diagnosed with hyperbilirubinemia. Variation within +/−1SD of the baseline mean of rSO_2_-C (%) and rSO_2_-R (%) was seen in 86.7% and 66.7% of the infants respectively in the post semi-upright (supine) position. In one infant, the rSO_2_-C (%) and rSO_2_-R (%) were more than 1SD below the mean in the post semi-upright (supine) position. Significant changes in cerebral and renal tissue oxygenation seen in the semi-upright position were not associated with lower gestational age, post-conception age, weight at discharge, or presence of anemia (Table 3). Anemia was recorded in 25% of infants with rSO_2_-C levels more than 1SD below the baseline mean in the semi-upright position as compared to 55.0% of infants with rSO_2_-C changes that were less than 1SD below baseline mean.

### Results of multivariate regression analysis

A multivariate regression model was used to identify whether the changes in rSO_2_-C (%) and rSO_2_-R (%) observed after repositioning from supine to the 45 degree semi-upright position persisted after adjustment for the variation in HR, RR, and SpO_2_ (%). It showed an association between repositioning of the infant from supine to the semi-upright position with decreased cerebral and increased renal tissue oxygenation (b = −0.056 +/− 0.008, P < 0.0001 and 0.092 +/− 0.008, P < 0.0001, respectively).

## Discussion

This study is the first that compared tissue oxygenation parameters in the recommended 45 degree semi-upright position in a car seat^12^ to those in the standard supine position^13^ in order to identify the repositioning-associated changes in the cerebral and renal tissue oxygenation of clinically stable preterm neonates. In the majority of the studied infants, changes in cerebral and renal tissue oxygenation seen after re-positioning to the 45 degree semi-upright position were within normal limit. Approximately one-third of studied infants showed dissimilar alterations in cerebral and renal tissue oxygenation in association with repositioning to the car seat. There was decreased cerebral tissue oxygenation with further normalization to the baseline levels and prolonged increase in renal tissue oxygenation despite repositioning to the supine position in a bassinet. It is uncertain what factors affect the difference in physiological response (change in tissue oxygenation) with respect to re-positioning of the infant from supine to the 45 degree semi-upright position. Conceivably, the inconsistent decrease in cerebral oxygenation is associated with adjustment of intracranial hemodynamics in the 45 degree semi-upright position^14^ and the ability of cerebral tissue to auto-regulate perfusion^15^ to meet tissue oxygen demand^16^. The increase in renal tissue oxygenation was unexpected because the semi-upright position is reported to be associated with an increase in the intra-abdominal pressure^17^,^18^, and decrease of abdominal perfusion pressure as well as filtration gradient^17^. It was previously shown that renal oxygenation can remain stable over a wide range of changes in renal blood flow^19^,^20^, and small increases of intra-abdominal pressure in mechanically ventilated adult patients do not affect renal perfusion and function^21^. Perhaps, the placement of stable preterm infants in the 45 degree semi-upright position does not imbalance renal perfusion, function, metabolic demand, or requirement for an increase in oxygen consumption by the renal tissue^22^.

In our study, renal FOE during the car seat testing was lower than in the baseline supine position. It is possible that the 45 degree semi-upright position is more favorable for renal tissue oxygenation of preterm born infants at the pre-discharge stage as compared to the generally used supine position. However, at this point in time, an explanation for such a phenomenon cannot be provided without further investigation of the renal function of infants in different body positions. The clinical value of the obtained results is reinforced by the determination of alterations in cerebral and renal tissue oxygenation in association with the body re-positioning not only in the aggregated data but also at the individual level^23^ and determination of effect-size of observed changes using the Z score that provides results independent from the sample size^24^.

One of the limitations of the present study is the observation in a car seat for only 60 minutes. It is possible that a longer duration in the semi-upright position could perhaps lead to a more manifest decrease in cerebral tissue oxygenation. However, the duration of observation in the 45 degree semi-upright position was allied with the hospital-based protocol for car seat testing adjusted to the time required for travel to homes of the discharged neonatal population. Another limitation is the non-detection of the sleep state of the infants during the measurement of tissue oxygenation. Although the infants were studied while they were spontaneously sleeping in a steady position (no cry or motor activity), there is still a risk for undetected variability in their sleep state, which may impact cerebral tissue oxygenation. In preterm infants aged 2–4 weeks, the quiet sleep state was found to be associated with higher levels of cerebral oxygenation as compared to the active sleep state^25^. The changes in cerebral tissue oxygenation associated with the sleep state were irrespective of the body position (supine versus prone). Regardless of the sleep state (active versus quiet), decreased levels of cerebral tissue oxygenation were recorded in the prone position as compared to supine. Such an association between changes in cerebral tissue oxygenation with respect to the body position and sleep state implies no significant interaction effect of the sleep state on the changes in cerebral oxygenation during re-positioning from the supine to prone position. No study has analyzed the role of sleep state in altering cerebral tissue oxygenation in preterm infants placed in the semi-upright position.

Although the current study is based on a small sample, features such as the very high number of simultaneously recorded measurements of tissue oxygenation and cardio-respiratory parameters and use of control data from the same subjects are the factors that suggest reliability of the obtained results. The study shows clinically insignificant alteration of cerebral and renal tissue oxygen saturation during re-positioning to the 45 degree semi-upright position in most of the stable preterm infants. However, the reduction in cerebral tissue oxygenation that was seen in approximately 30% of the tested neonates is an important finding since about 85% of the rSO_2_-C readings are from the cortical tissue^26^, which underlies the neurocognitive deficits seen in the preterm born infants^27^. Any reduction in cerebral oxygen content regardless of clinical stability, can be a potential cause for increase in de-oxygenation related pathology in very preterm born infants. Further research is required to elucidate and/or validate the role of pre-discharge cerebral tissue oxygenation monitoring in the development of recommendations for optimal body positioning of preterm born infants whether in a car seat or at home.

## Methods

The study protocol and parental consent forms were approved by the Institutional Review Board at Rutgers Robert Wood Johnson Medical School. Immediately prior to the pre-discharge car seat testing of stable preterm born infants (without congenital malformations, severe brain pathology or apnea of prematurity), maternal informed consent was obtained for the participation of their infants in this study. All the study methods were performed in accordance with the relevant guidelines and regulations.

We used near-infrared spectroscopy (NIRS) equipment (INVOS 5100B, Somanetics Corporation, Michigan, USA) to record cerebral (rSO_2_-C %) and renal (rSO_2_-R %) tissue oxygenation at sampling intervals of 5 seconds. One probe was placed on the forehead of the infant and another to the right of midline (T10-L2 posterior flank), which is the thoraco-lumbar projection of the right kidney. All the NIRS measurements were performed during spontaneous sleep in a steady position. To prevent sensor movement/detachment and artifacts during the repositioning, VELCRO® straps (from the phototherapy eye patches) were used to support the sensor placed on the forehead and bio-occlusive dressing was utilized to secure the sensor placed over the posterior flank.

NIRS is a non-invasive technique used for the continuous measurement of regional tissue oxygenation in preterm infants^28^,^29^. Because of the concern regarding biological variability of NIRS recorded oxygen saturations in different tissues^30^, we compared oxygenation parameters obtained using trend monitoring in the same subject under different body positioning conditions^31^. Heart Rate (HR), Respiratory Rate (RR) and pulse oximetry measured SpO_2_ (%) required for the Infant Car Seat Challenge (ICSC)^11^, were recorded simultaneously with the GE Dash 4000 (GE Healthcare, Wisconsin, USA). The GE Dash 4000 was also used to measure systolic (SBP), diastolic (DPB), and mean blood pressure (MBP) approximately 30 minutes prior to the car seat challenge. For newborn infants, the GE DASH 4000 monitor displays a RR of 0–200 breaths per minutes (bpm) with an accuracy of +/−3 bpm, blood pressure is displayed in the range of 30–135 mmHg for SBP, 10–110 for DBP, and 10–125 for MBP, and SpO_2_ ranges from 70–100% and from 50–65% with an accuracy of +/−2% and 3%, respectively.

Tissue oxygenation (rSO_2_-C and rSO_2_-R) and cardio-respiratory parameters (HR, RR, and SpO_2_) were interfaced by matching exact times of measurements recorded every 5 seconds. We calculated the cerebral and renal fractional oxygen extraction ratio (FOE-C and FOE-R) that represents the proportion of delivered oxygen utilized by tissue [FOE = (SaO_2_–rSO_2_)/SaO_2_]^32^. Hypotension was defined as a MBP less than 30 mmHg^33^, bradycardia as HR ≤ 80 beats/minute^34^, and hypoxia as SpO_2_ of ≤80%^35^.

Data were collected in 3 positions: (i) supine (for 30 minutes) in a bassinet, (ii) 45 degree semi-upright (for 60 minutes) in a car seat, and (iii) post-semi-upright supine (for 30 minutes) in a bassinet. Infants were studied between feedings and left undisturbed during the ICSC testing performed by a trained NICU nurse who monitored the HR, RR, and SpO_2_. To determine failure of the 60-min ICSC testing, AAP criteria^12^ were used: (a) any desaturation <90% lasting >10 seconds, (b) apnea ≥20 seconds, (c) bradycardia ≤80 beats-per-minute, and (d) any alteration in vital signs, work-of-breathing or respiratory distress. The studied infants passed the ICSC test without any interaction of the research team with the staff that evaluated the ICSC test.

In addition to the tissue oxygenation and cardiorespiratory parameters, demographic and clinical data, that included gender, race/ethnicity, gestational age and corrected gestational age, birth weight and discharge weight, clinical conditions during hospitalization, clinical status and results at or close to discharge were collected. Anemia was defined as hemoglobin (Hb) and hematocrit (Hct) levels <10.6 g/dL and <33%, respectively^36^.

### Statistical analysis

Chi-square test was used to compare differences in the categorical variables. Repeated measures analysis of variance and Tukey's honestly significant differences (HSD) were used to compare the mean levels of tissue oxygenation and cardio-respiratory measurements as follows: (i) baseline supine to semi-upright and (ii) baseline supine to post-semi-upright supine. Comparisons were made for the whole group and each participant individually. A significant difference with small effect-size is to be expected due to the large number of recordings (every 5 seconds for approximately 120 minutes for each subject). In order to identify meaningful data from comparison of the recorded parameters for the cohort and for each individual subject with respect to change in body position, Z scores were calculated. Z scores are reported as the number of SDs by which measurements in the semi-upright position and after repositioning to the supine position differed from the baseline mean levels. The magnitude of effect was identified by a change in tested parameters below or above 1SD of the mean at the baseline supine position. Parameters were classed as indistinguishable if the mean levels of the tested parameters in the entire group or each subject were within 1SD from those obtained in the supine position. Since the pathological threshold for oxygen content of cerebral and renal tissue in preterm born infants is not known, cerebral and renal tissue desaturation was defined as decrease in rSO_2_ (%) of more than 1SD, which is a reasonable cutoff for defining clinical significance^37^.

Multiple regression analysis was performed to ascertain the role of re-positioning in the altering of the rSO_2_-C and rSO_2_-R after controlling for the variability in HR, RR, and SpO_2_ (%). In addition, changes in rSO_2_-C and rSO_2_-R were analyzed with respect to the gestational and post-conception age, and diagnosis of anemia of prematurity. Data are presented as mean, standard deviation (SD), range, Z-score, percentage (%), and regression coefficient (b) +/−standard error (Std. Err) of regression coefficient. A difference at a two-tailed P value of less than 0.05 was considered statistically significant. Statistical analysis was performed using STATISTICA 12.0 for Windows (StatSoft Inc, Oklahoma, USA).

## Author Contributions

A.P. designed the study, analyzed and interpreted the data, and drafted the manuscript. R.M. conceptualized the study, collected the data, and revised the manuscript critically for important intellectual content. All authors reviewed the manuscript.

## Figures and Tables

**Table 1:** Demographic and clinical characteristics of the studied infants (n = 15)

Characteristics	Mean +/− SD, range
Birth weight (Mean +/− SD, range, grams)	1696 +/− 394 (800–2250)
Discharge weight (Mean +/− SD, range, grams)	2142 +/− 293 (1750–2885)
Gestational age (Mean +/− SD, range, weeks)	31.4 +/− 3.4 (25–36)
Post-conception age (Mean +/− SD, range, weeks)	35.3 +/− 1.4 (33–38)
Race/ethnicity (n, %)	
White	40.0% (n = 6)
Black	20.0% (n = 3)
Hispanic or Other	40.0% (n = 6)
Gender (Male, n, %)	33.3% (n = 5)
Vaginal delivery (n, %)	80% (n = 12)
Hemoglobin (Mean +/− SD, range, g/dL)	11.0 +/− 2.3 (7.9–17)
Hematocrit (Mean +/− SD, range, %)	33.8 +/− 6.8 (22.4–50)
Systolic Blood Pressure	76.7 +/− 6.0 (67–91)
Diastolic Blood Pressure	42.2 +/− 5.2 (32–50)
Mean Blood Pressure	52.9 +/− 4.8 (43–58)

**Table 2:** Comparison of tested parameters with respect to the preterm infants' body positioning (Mean +/− SD, Z- score)

Parameters	Positioning		
	Supine baseline (1)	Semi-upright (2)	Supine after repositioning (3)
**HR (bits/min)**	153.2 +/− 14.6	152.1 +/− 12.7	148.7 +/− 14.4^1–3^(*)
**Z- score**		−0.075^1–2^	−0.308^1–3^
**RR (n/per min)**	44.7 +/− 10.6	49.1 +/− 14.9^1–2^(*)	43.5 +/− 11.9
**Z- score**		0.415^1–2^	−0.113^1–3^
**SpO2 (%)**	98.5 +/− 2.2	98.1 +/− 2.0	98.7 +/− 1.5
**Z- score**		+0.182^1–2^	+0.091^1–3^
**rSO2C (%)**	69.1 +/− 5.8	67.7 +/− 7.5^1–2^(*)	68.9 +/− 7.1
**Z- score**		−0.241	+0.091^1–3^
**rSO2R (%)**	67.6 +/− 9.5	70.1 +/− 9.8^1–2^(*)	70.8 +/− 12.6^1–3^ (*)
**Z- score**		+0.263^1–2^	+0.337^1–3^
**FOEC**	0.298 +/− 0.064	0.309 +/− 0.078^1–2^(*)	0.301 +/− 0.074
**Z- score**		+0.172^1–2^	+0.047^1–3^
**FOER**	0.312 +/− 0.101	0.285 +/− 0.102^1–2^(*)	0.282 +/− 0.131^1–3^ (*)
**Z- score**		−0.267^1–2^	−0.297^1–3^

*P < 0.00001.

**Table 3:** Comparison of the infants' clinical parameters in association with the changes in rSO_2_-C and rSO_2_-R after re-positioning to the semi-upright position

Parameters	Changes in rSO2-C and rSO2-R in semi-upright position					
	rSO_2_-C			rSO_2_-R		
	Within SD (n = 9)	Below SD (n = 4)	Above SD (n = 2)	Within SD (n = 9)	Below SD (n = 1)	Above SD (n = 5)
Anemia (%)	55.6%	25%	50%	55.6%		20%
GA* (M +/− SD, wks)	31.6 +/− 2.9	30.7 +/− 4.5	32.0 +/− 1.4	30.1 +/− 3.1	36.0	32.8 +/− 1.6
Age ** (M +/− SD, wks)	35.4 +/− 1.2	35.2 +/− 2.2	34.5 +/− 0.7	35.2 +/− 1.5	38.0	34.8 +/− 0.4
Weight *** (M +/− SD, g)	2232 +/− 317	2024 +/− 193	1975 +/− 318	2147 +/− 382	2275	2106 +/− 39

*Gestational age (GA);

**Post-conception age (Age);

***Weight at discharge (Weight).
